# Karyotype structure and chromosome fragility in the grass *Phleum echinatum* Host

**DOI:** 10.1007/s00709-014-0681-5

**Published:** 2014-07-24

**Authors:** Aleksandra Grabowska-Joachimiak, Adam Kula, Dorota Gernand-Kliefoth, Andrzej J. Joachimiak

**Affiliations:** 1 Department of Plant Breeding and Seed Science, University of Agriculture in Cracow, Łobzowska 24, Cracow, 31-140 Poland; 2 Leibniz-Institute of Plant Genetics and Crop Plant Research (IPK), Gatersleben, 06466 Germany; 3 Department of Plant Cytology and Embryology, Institute of Botany, Jagiellonian University, Gronostajowa 9, Cracow, 30-387 Poland

**Keywords:** *Phleum echinatum*, Fragile sites, FISH, rDNA, Interstitial telomeric sequences, Chromosome fusions

## Abstract

*Phleum echinatum* Host (2*n* = 2*x* = 10) is an annual Mediterranean species which differs from other representatives of the genus *Phleum* by reduced chromosome number, asymmetric karyotype and unusually high amount of DNA in the genome. Chromosomes of this plant were studied using conventional acetic-orcein staining and fluorescence in situ hybridization (FISH). FISH showed the major 35S ribosomal DNA (rDNA) site at the secondary constriction of satellite chromosome (3) and the minor 35S rDNA site near 5S rDNA cluster in the monobrachial chromosome 5. Telomeric repeats were detected at all chromosome ends within secondary constriction in satellited chromosome 3 and at the centromeric regions of chromosomes 1 and 2. Intrachromosomally located telomeric repeats are probably traces of chromosomal rearrangements that have shaped *P.echinatum* genome; they were prone to breakage which was manifested in chromosome fragmentation. The most distinct telomeric signals, suggesting massive amplification of interstitial telomeric sequences (ITRs), were observed at the nucleolar organizer region (NOR) of the third chromosome pair. Double FISH confirmed co-localization of telomeric and 35S rDNA repeats in this locus characterized by the biggest fragility in the karyotype. Fragile sites of *P.echinatum*, composed of amplified telomeric repeats, may bear a resemblance to metazoan rare fragile sites enriched in microsatellite repeats.

**Electronic supplementary material:**

The online version of this article (doi:10.1007/s00709-014-0681-5) contains supplementary material, which is available to authorized users.

## Introduction

*Phleum echinatum* Host is an annual Mediterranean species of timothy belonging to the section *Phleum*. Its somatic chromosome number 2*n* = 2*x* = 10, established by Ellestrom and Tijo in 1950, was confirmed after 55 years (Kula 2005). The basic chromosome number *x* = 5 in *P.echinatum* differs from the number *x* = 7 occurring in all other representatives of the genus *Phleum* (Joachimiak and Kula 1993, 1996; Joachimiak 2005; Stewart etal. 2009). Moreover, the species shows some other untypical features—it is characterized by distinct intrachromosomal asymmetry of the karyotype, much bigger chromosomes and a higher DNA content in the genome (Cx = 3.64 pg vs. Cx = 1.34–1.69 pg in other species) (Sliwinska etal. 2003; Joachimiak 2005; Kula 2005). All these observations suggest that *P.echinatum* shows a derived, highly differentiated genome, which was shaped as a result of dysploid reduction and increase in genomic DNA amount.

Kula (2005) noted that the majority of the metaphase plates of *P.echinatum* which were observed contained fragments of chromosomes. Significantly, such chromosome fragmentation was not observed in any of other *Phleum* species analyzed by him. It could suggest that in *P.echinatum* karyotype, fragile sites may occur. Fragile sites are manifested as non-random breaks and gaps on metaphase chromosomes (Casper etal. 2002). So far, chromosome lesions caused by the occurrence of such points in chromosomes have been most frequently observed in animals. Various ranges of this phenomenon were described in, e.g. insects, rodents and primates (Toledo etal. 2000; Ruiz-Herrera etal. 2002). In humans, fragile sites in autosomes are frequently involved in chromosomal rearrangements in cancer cells (Hellman etal. 2002), whereas fragility of the X chromosome is responsible for the most common familial form of mental retardation (Kaufmann and Reiss 1999). In plants, non-random chromosome breaks are definitely less frequent and almost exclusively restricted to interstitial 35S ribosomal DNA (rDNA) sites, and this phenomenon was analyzed in more detail in *Lolium* (Huang etal. 2008, 2009).

More precise studies of *P.echinatum* concerning location of repetitive sequences (rDNA, telomeric sequences) have not been performed yet. Because of reduced chromosome number, much attention should be paid to possible traces of chromosomal rearrangements in this species. Moreover, it should be explained whether observed chromosome fragmentation is accidental or the breaks occur in some particular sites. Our main objectives were (i) to localize chromosome breaks; (ii) to show the location of chromosomal sites of 5S rDNA, 35S rDNA, and telomeric sequences; and (iii) to analyze the *P.echinatum* karyotype and its chromosome fragile sites more precisely.

## Materials and methods

### Plant material

The plant material was collected from a natural stand in Sicily and delivered by the Botanical Garden in Bydgoszcz, Poland. In both cases, it came from the area between the town of Ficuzza and the Rocca Busambra foothills (1,613 m.a.s.l.) situated in the north-west of Sicily. For comparison, during identification of the material, original herbarium cards of *P.echinatum* Host from the Botanical Garden of Palermo were used.

### Chromosome preparations

The chromosome preparations were obtained from root-tip meristems of 20 specimens. Excised root tips were incubated in 8-hydroxyquinoline for 4 h at room temperature, rinsed in distilled water and fixed in absolute ethanol/glacial acetic acid (3:1) for 24 h. Then, the material was stained with 2 % acetic-orcein according to Marciniuk etal. (2012) or softened and prepared for fluorescence in situ hybridization (FISH) according to Gernand etal. (2007). Chromosome lengths were calculated on the basis of measurements performed on digitally captured chromosomes (NIS-elements software, Nikon).

### DNA probes and fluorescence in situ hybridization

The *Arabidopsis thaliana*-derived clone pCT4.2 (Campell etal. 1992) and BAC clone (EMBL accession no. AF167571) were used as 5S and 35S rDNA probes, respectively. An *Arabidopsis*-type telomere probe was generated by PCR amplification according to Ijdo etal. (1991).

In situ hybridization sequences were labeled by nick translation or PCR with digoxigenin-11-dUTP or biotin-16-dUTP. FISH on the squashed root tips was performed as described by Houben etal. (2001). Briefly, 20 ng of each probe was applied per slide. Hybridization sites of digoxigenated and biotinylated probes were immunodetected either by rhodamine-conjugated anti-digoxigenin sheep antibodies and rhodamine anti-sheep antibody for signal amplification or Alexa488-conjugated streptavidin and FITC-conjugated anti-streptavidin antibodies, respectively. FISH preparations were mounted and counterstained in Vectashield (Vector Laboratories), containing 2 μg/ml of DAPI. Epifluorescence signals were recorded electronically with a cooled charge-coupled device camera (ORCA-ER Hamamatsu). The image superimposition was performed with Adobe Photoshop 6.0.

## Results

### Chromosome number and morphology

**Fig.1:**
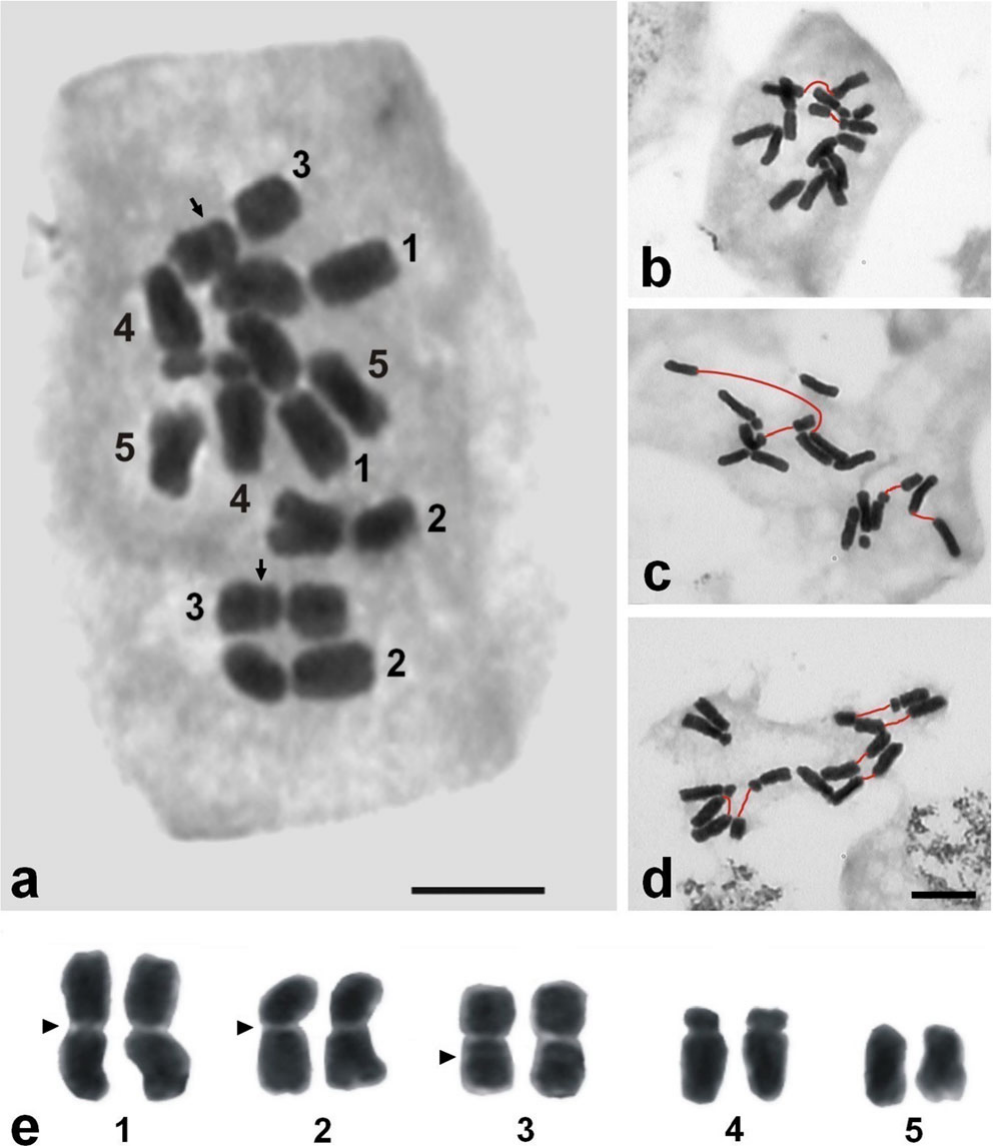
Chromosomes of *Phleum echinatum*. **a** Complete metaphase plate with 10 unfragmented chromosomes. *Arrows* point to the secondary constriction of satellite chromosomes (3). **b**–**d** Metaphase plates with chromosome breaks (*red*). **e** Conventional karyotype (2*n* = 10). *Arrowheads* point to the fragile sites. Bar, 5 μm

Among the 69 conventionally stained metaphase plates, only three plates had 10 unfragmented chromosomes (Fig. 1a). In the other plates, 11 to 16 chromosomes and their fragments were found (see supplementary material). Having accepted the karyotype structure presented by Ellestrom and Tijo (1950), an interpretation of particular metaphases with chromosome breaks was possible (Fig. 1b–d). *P.echinatum* karyotype contained two pairs of long metacentrics (8.93 and 7.22 μm), a pair of submetacentric satellited (SAT) chromosomes (6.72 μm), one pair of medium-sized subtelocentric chromosomes (5.58 μm) and one pair of the smallest in the karyotype telocentric chromosomes (4.18 μm) (Fig. 1e).

Detailed analysis of the metaphase plates with fragmented chromosomes revealed the presence of three fragile sites in the karyotype of *P.echinatum*. They were the secondary constriction of SAT chromosome (3) and the primary constrictions in the two longest chromosomes (1 and 2) (Fig. 1e). In only one of the analyzed metaphase plates, a chromosome lesion was observed in a different locus (lesion at centromere of chromosome 4).

### FISH mapping of rDNA and telomeric repeats

**Fig.2:**
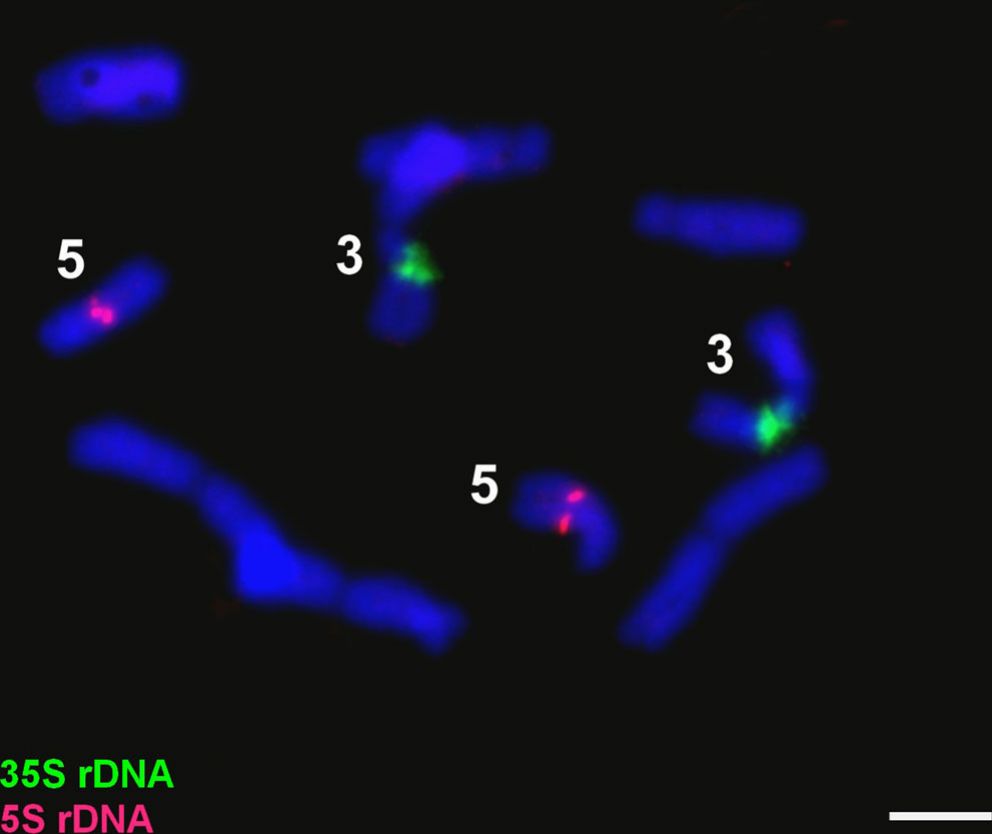
Double FISH with rDNA probes on *P.echinatum* metaphase chromosomes: 35S rDNA (*green signals*) in satellite chromosomes (3) and 5S rDNA (*red signals*) in telocentric chromosomes (5). Bar, 5 μm

**Fig.3:**
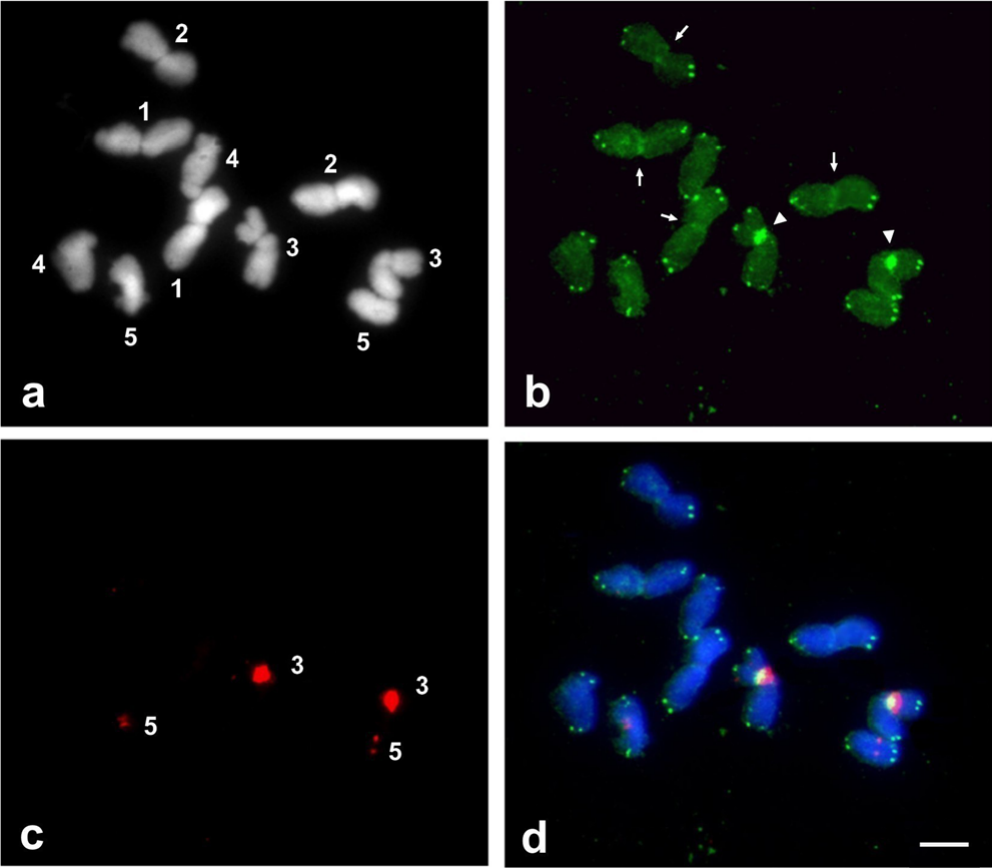
FISH mapping of telomeric (T_3_AG_3_) and 35S rDNA sequences to metaphase chromosomes of *P.echinatum*. **a** The DAPI-stained and numbered chromosomes. **b** FISH signals of telomeric repeats. *Arrowheads* point to the massive accumulation of interstitial telomeric sequences (ITRs) within the secondary constriction of satellite chromosomes (3). *Arrows* point to telomeric signals located at the centromeric regions of chromosomes 1 and 2. **c** FISH signals of 35S rDNA sequences located within satellite chromosomes (3) and telocentric chromosomes (5). **d** Double FISH with 35S rDNA (*red*) and telomeric (*green*) probes; Note the co-occurrence of telomeric repeats and 35S rDNA within the secondary constriction of satellite chromosomes (3). Bar, 5 μm

In all the preparations, two solid 35S rDNA signals were observed within satellite chromosomes (3), whereas 5S rDNA signals were detected in the middle of the two telocentric chromosomes (5) (Fig. 2). In some preparations, minor 35S rDNA sites were observed in these chromosomes (Fig. 3c,d).

**Fig.4:**
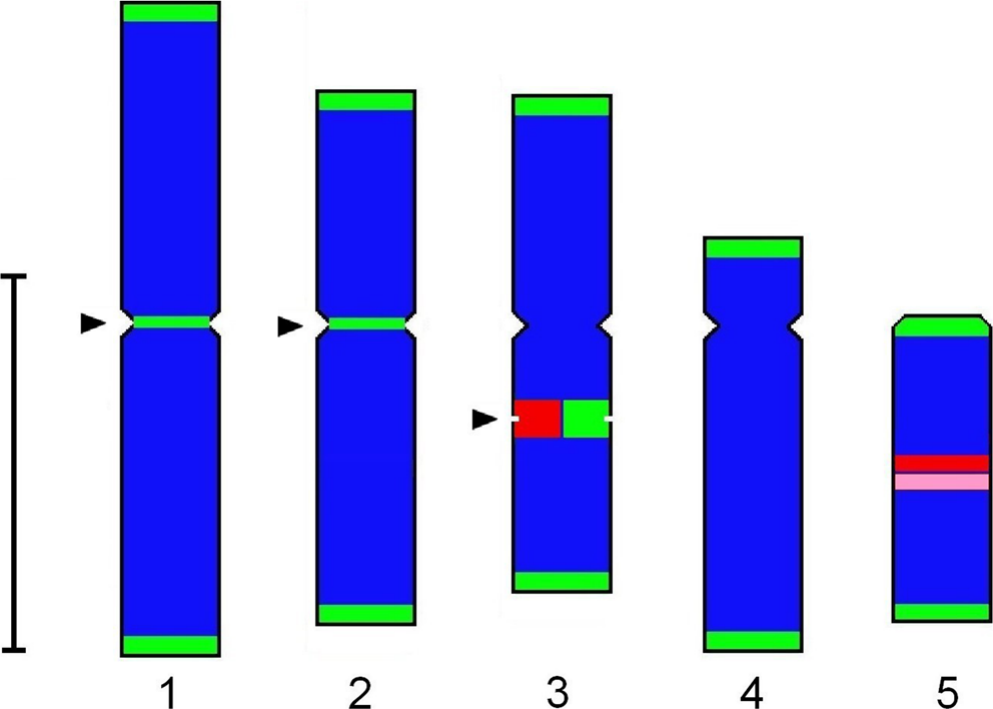
Idiogram of *P.echinatum* chromosomes with hybridization sites indicated: telomeric sequences (*green*), 35S rDNA (*red*) and 5S rDNA (*pink*). *Arrowheads* point to the fragile sites. Bar, 5 μm

Telomeric signals were revealed at the ends of all *P.echinatum* chromosomes (Fig. 3b). Furthermore, interstitially located TTTAGGG repeats were detected in the first three chromosome pairs: in centromeric regions of chromosomes 1 and 2 and within the secondary constriction of satellite chromosomes (3) (Fig. 3a,b). The signals within the secondary constrictions of satellite chromosomes were much bigger than these at the chromosome ends. Telomeric sequences in these sites co-occur with 35S rDNA (Fig. 3d). Interestingly, nucleolar organizer regions (NORs) enriched with these two sequences were most fragile in the karyotype, and during prophase and early metaphase, they were usually strongly decondensed (data not shown). The location of all the analyzed sequences and the fragile sites in *P.echinatum* chromosomes are presented in Fig. 4.

## Discussion

Changes in basic chromosome numbers played an important role in the evolution of grasses (Kellogg 1998; Salse etal. 2009; Devos 2010). The most reduced chromosome number in this group of plants (2*n* = 2*x* = 4) occurs in *Zingeria biebersteiniana* and *Colpodium versicolor* (Bennett etal. 1995; Kim etal. 2009; Ruffini Castiglione and Cremonini 2012). Most probably, the direct ancestor of both species, just like the ancestor of *P.echinatum*, had the basic chromosome number *x* = 7 (*p* = 7 according to Peruzzi 2013). Our research has confirmed the earlier data concerning both the number and morphology of chromosomes in *P.echinatum* (Ellestrom and Tijo 1950; Kula 2005). Moreover, it has revealed that apart from the secondary constriction in satellite chromosome, 35S rDNA may occur in this species also in the central part of the shortest telocentric chromosome.

The occurrence of fragile sites in the three largest chromosomes seems the most interesting feature of the *P.echinatum* karyotype. They all contain intrachromosomal telomeric sequences in sites preferential for heterochromatin. It is well known that both centromeric regions and NORs are domains enriched with heterochromatin (Joachimiak etal. 1997; Guerra 2000; Amor etal. 2004; Henikoff and Dalal 2005). According to Ruiz-Herrera etal. (2008), heterochromatic intrachromosomal telomeric repeats (het-ITRs) are particularly prone to breakage in mammalian genomes.

In the secondary constriction of chromosome 3, the most fragile site, 35S rDNA is accompanied by telomeric repeats. The NOR regions were found to exhibit fragility and mobility in many plant species (Thomas etal. 2001; Huang etal. 2008; Raskina etal. 2008). It could be linked with the mobility of rDNA per se or the activity of the transposable elements located near or within rDNA clusters (Schubert and Wobus 1985; Gernand etal. 2007; Raskina etal. 2008). The co-occurrence of rDNA and telomeric repeats in the secondary constriction of chromosome 3 in *P.echinatum* might be an extra factor destabilizing this site.

In mammalian genomes, the length of interstitial telomeric sequences (ITRs) (and other microsatellite repeats) is one of the factors leading to genome instability (Lin and Yan 2008). It has been suggested that from many ITRs, only large blocks of telomeric repeats (spanning several hundred kb) are involved in chromosome breakage, whereas instability of short ITRs is more controversial (Lin and Yan 2008; Ruiz-Herrera etal. 2008). ITRs observed in *P.echinatum* definitely contain a high number of repeats because FISH performed on condensed chromosomes cannot detect target loci smaller than 10 kb. It is worth mentioning that the most fragile site in the *P.echinatum* karyotype was also characterized by the largest accumulation of telomeric repeats.

As far as the origin is concerned, ITRs in *P.echinatum* are most probably remnants of evolutionary chromosomal fusions which led to the reduction in chromosome number in this species. The large size of the three chromosomes in which they occur also suggests that they could have been shaped this way. The secondary constriction of chromosome 3, where telomeric sequences are located near 35S rDNA, seems particularly interesting. According to Raskina etal. (2008), the occurrence of interstitially located 35S rDNA clusters and traces of telomeric sequences inside 35S rDNA is unquestionable indicators of chromosome rearrangements. Telomeric sequences in this place could be traces of chromosome healing provoked by earlier breaks within rDNA. It is well known that double-stranded breaks provide templates for new telomeres (Bolzan and Bianchi 2006). Later, chromosome fragments containing rDNA, ended with telomeres formed in this way, might have been subject to a fusion, creating chromosome 3 of *P.echinatum* within which further amplification of telomeric sequences may have taken place. It has been suggested that ITRs resulting from fusion can undergo amplification through various mechanisms (unequal crossing over, replication slippage, conversion-like mechanisms, and rolling circle replication) (Lin and Yan 2008). The other ITRs of *P.echinatum* were located in the regions of centromeres of chromosomes 1 and 2. Large, similarly located sequences of telomeric origin were found in the karyotype of the potato (Tek and Jiang 2004). According to the authors, there are traces of earlier chromosome fusions.

Our study has fully confirmed the supposition that the *P.echinatum* karyotype is a product of dysploid reduction caused by complex chromosome rearrangements followed by the loss of centromeres. These changes were accompanied by massive amplification of some sequences, which led to doubling the DNA amount in the basal genome of this species. It is interesting because the genus *Phleum* is known to have small genome among Pooideae, which is close to the estimated ancestral 1.3 pg (Kellogg and Bennetzen 2004). The research conducted has shown so far that the increases in basic genome size occur predominantly by episodic transposon bursts, often associated with chromosome rearrangements or provoked by hybridity (Lonnig and Saedler 2002; Bennetzen 2005).

The closest diploid relatives of *P.echinatum* (belonging to the *Phleum alpinum* group) are perennial taxa with medium-sized chromosomes, symmetric karyotype and small genome size (Joachimiak and Kula 1996; Joachimiak 2005; Stewart etal. 2011). The stomata of *P.echinatum* (~46 μm) are bigger than those in di- and even tetrapoid representatives of *P.alpinum* group (Kula 2005) and in hexaploid *Phleum pratense* (Joachimiak and Grabowska-Joachimiak 2000). Watanabe etal. (1999) suggested that the reduction in chromosome number, increase in mean chromosome length and karyotype asymmetry are correlated in plants with the change in habit from perennial to annual. According to these authors, lowering of chromosome number results in a reduction of total chromosome length (genome size) and cell size, which favours shortening of cell cycle in annuals. *P.echinatum* is characterized by annual habit and significantly increased genome and cell size, then it does not fit this pattern. This may suggest the involvement of other factors (e.g. hybridity) in the origin of this species. More detailed explanation of evolutionary processes which shaped its chromosomes requires further research.

## Electronic supplementary material

Below is the link to the electronic supplementary material.

ESM 1 (JPEG 3861 kb)
