# Supplementary figures and images for: Karyotype structure and chromosome fragility in the grass Phleum echinatum Host
Source: Protoplasma. 2014 Jul 24;252(1):301–6. doi: 10.1007/s00709-014-0681-5 (PMC4287660; doi:10.1007/s00709-014-0681-5)

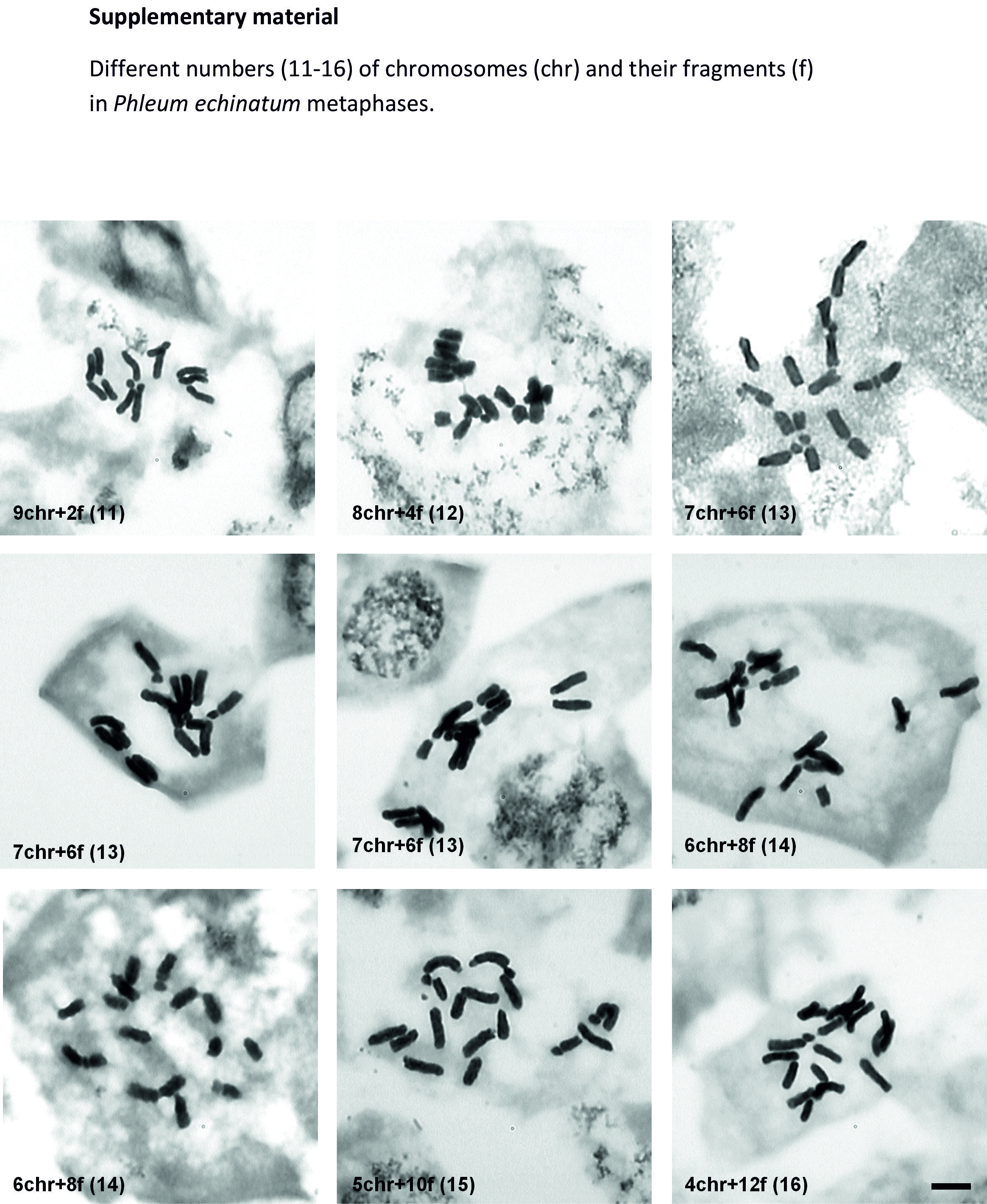

Supplement: Supplementary file 1 — (JPEG 3861 kb) [file 709_2014_681_MOESM1_ESM.jpg]
